# Equi–size nesting of Platonic and Archimedean metal–organic polyhedra into a twin capsid

**DOI:** 10.1038/s41467-020-17989-6

**Published:** 2020-08-14

**Authors:** Hongmei Gan, Na Xu, Chao Qin, Chunyi Sun, Xinlong Wang, Zhongmin Su

**Affiliations:** National & Local United Engineering Laboratory for Power Batteries, Key Laboratory of Polyoxometalate Science of Ministry of Education, Northeast Normal University, Changchun, Jilin China

**Keywords:** Coordination chemistry, Self-assembly

## Abstract

Inspired by the structures of virus capsids, chemists have long pursued the synthesis of their artificial molecular counterparts through self–assembly. Building nanoscale hierarchical structures to simulate double-shell virus capsids is believed to be a daunting challenge in supramolecular chemistry. Here, we report a double-shell cage wherein two independent metal–organic polyhedra featuring Platonic and Archimedean solids are nested together. The inner (3.2 nm) and outer (3.3 nm) shells do not follow the traditional “small vs. large” pattern, but are basically of the same size. Furthermore, the assembly of the inner and outer shells is based on supramolecular recognition, a behavior analogous to the assembly principle found in double-shell viruses. These two unique nested characteristics provide a new model for Matryoshka–type assemblies. The inner cage can be isolated individually and proves to be a potential molecular receptor to selectively trap guest molecules.

## Abstract (web summary)

Supramolecular constructs that mimic complex biological assemblies are synthetically challenging. Here, the authors present a double-shell cage wherein two independent metal-organic polyhedra are nested together in a manner analogous to that found in double-shell virus capsids.

## Introduction

The attractiveness of molecular coordination cages^1–5^, sometimes termed as metal–organic polyhedra (MOPs)^6^, lies in their structural resemblance to natural living organisms, for example, the icosahedron is reminiscent of spherical viruses^7,8^ and rhombic dodecahedron is akin to ferritin^9^. Therefore, the research on these capsid polyhedral structures could help scientists to design and develop more rational antiviral strategies. Currently, self-assembly of metal–organic capsules by taking advantage of the rational and judicious selection of reaction components has been well-established, represented by the prominent work of Stang^10,11^ and Fujita and co-workers^12–14^; however, it is still a daunting challenge for chemists to produce hierarchical polyhedral capsids that consist of well-defined sub-shells similar to the onion-type arrangements.

**Fig. 1:**
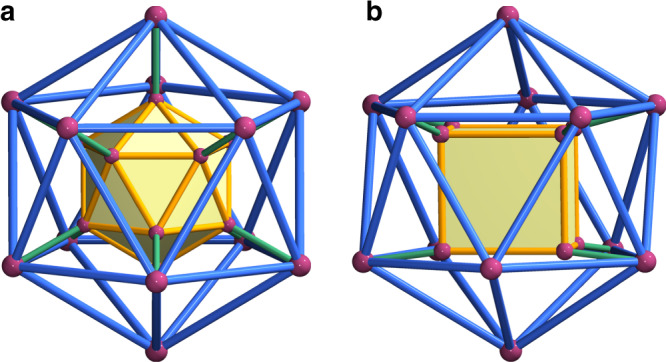
Two known endohedral models. The inner (marked in orange) and outer (marked in blue) cages of different sizes, whether they have the same (**a**) or different (**b**) geometry, are interconnected by covalent linkages (marked in green).

Double-shell architectures are special in chemistry and rare in non-biological chemistry although frequently occur naturally in the form of spherical virus capsids^15–17^. Artificial synthesis of such hierarchically organized cage-within-cage structures should be able to further our understanding of the interaction behavior between inside and outside shells of virus capsids at the molecular scale. And equally importantly, it may provide a new class of functional supramolecular hosts. However, progress in this area has been frustrated by the absence of the underlying assembly principles. The design synthesis of double-shell architectures is still an elusive target for researchers. Unlike single-wall molecular aggregates^18–21^, the intrinsic structural particularity of double-shell MOPs sets a higher requirement for building components, especially for organic ligands in that how to reinforce structural subdivision is a key factor to be considered in the process of self-assembly. Because of this, with the exception of a few sporadic reports on metal–organic frameworks (MOFs) containing double-shell subunits so far^22–26^, double-shell macromolecular cages are much more rarely observed in the literature, with Pd-involved self-assembly being the commonest. This series of sphere-in-sphere assemblies with a general formula of Pd_24_L_24_ are pioneered by Fujita and colleagues by selecting a tethered ligand on purpose^27^, and are developed in the groups of Li^28^ and Mukherjee^29^ by precise mapping of ligand coordination sites. More recently, Schmitt et al.^30^ have reported the synthesis of ultra-large coordination cages that are composed of multiple smaller sub-cages. The basic attributes of these existing cases have been investigated and schematically shown in Fig. 1. First, the inner and outer cages are not absolutely independent but interconnected by covalent metal–ligand contacts. This behavior is different from what has been seen in living systems wherein only supramolecular interactions exist between the shells. Second, the inner and outer cages, irrespective of their geometrical configuration, always have an obvious difference in size, the smaller inside, and the larger outside. This, then, raises the crucial question of whether there may exist entirely new nesting topologies that do not comply with the aforesaid rules. If we can find such an example, it will provide a valuable structural paradigm for this infant family, and more importantly, such a finding might shed light on the hidden nesting mechanism that guides the synthesis of the highly complicated structures, thus taking an important step towards a closer mimic of the complex biological self-assembly.

Our serendipitous experimental outcome addresses this issue. Herein we present an elegant double-shell cage, which was formulated as (NH_2_Me_2_)_24_{[(V_5_O_9_Cl)_6_(TATB)_8_][V_12_O_12_(TATB)_8_(HSO_4_)_12_]}∙(CH_3_OH)_16_ by elemental microanalysis, thermogravimetric analysis (TGA) (Supplementary Fig. 1), and a single-crystal X-ray diffraction study (hereafter **1** for short, H_3_TATB = 4,4',4"-s-triazine-2,4,6-triyl-tribenzoic acid, DMF = N,N-dimethylformamide). In **1**, a [(V_5_O_9_Cl]_6_(TATB)_8_] octahedron perfectly nests in a [V_12_O_12_(TATB)_8_(HSO_4_)_12_] cuboctahedron. The double-shell MOP exhibits two distinguishing characteristics. One is that the assembly of the inner and outer shells is guided by non-covalent interactions (herein refers to π...π supramolecular recognition), a manner reminiscent of the analogous assembly principle found in double-shell virus capsids; the other is that the nesting occurs in two nearly equi–sized polyhedra, one 32 Å and the other 33 Å—an almost impossible case in a normal sense, making the endohedral degree to the extreme. What is even more interesting is that the inner polyoxovanadate-based cage, namely, [(V_5_O_9_Cl)_6_(TATB)_8_]^12−^ (**1a**), has been isolated individually. **1** and **1a** can selectively adsorb cationic dye molecules due to their anionic nature. In addition, **1a** is capable of serving as a supramolecular receptor to selectively encapsulate guest molecules (C_60_ and anthracene) owing to its large void space. Temperature-dependent magnetic susceptibilities for **1** and **1a** were also studied, and the weak ferromagnetic coupling interactions within the double-shell cage or the independent interior cage have been observed.

## Results

### Synthesis and description of double-shell cage

Compound **1** was synthesized by the solvothermal reaction of NaVO_3_, VOSO_4_, VCl_3_ and H_3_TATB in DMF:CH_3_CN:CH_3_OH (4:1:1 v/v) at 130 °C for 48 h (see the “Methods” section for a detailed synthesis procedure). The sensitivity of the synthesis to temperature needs to be considered; the product is not available below 130 °C, indicating that **1** is the thermodynamically favored outcome. The phase purity of the bulk products was confirmed by comparison of the observed and calculated powder X-ray diffraction (PXRD) patterns (Supplementary Fig. 2). Single-crystal X-ray diffraction analysis revealed that **1** crystallized in the tetragonal space group *I4/m* (Supplementary Table 1). The anionic moiety is composed of two independent coordination cages with the formula [(V_5_O_9_Cl)_6_(TATB)_8_][V_12_(TATB)_8_(HSO_4_)_12_]^24−^. The 24 negative charges are balanced by twenty-four dimethylamine cations (H_2_NMe_2_)^+^ (the byproduct of in situ decomposition of DMF molecules). The existence of (H_2_NMe_2_)^+^ is confirmed by IR spectrum (Supplementary Fig. 3). Scanning electron microscopy with energy-dispersive X-ray spectroscopy (SEM–EDX, Supplementary Fig. 4) and X-ray photoelectron spectroscopy (XPS) (Supplementary Fig. 5, 6) confirmed the sample purity and the oxidation states of vanadium atoms. Overall bond valence sum (BVS)^31^ calculations indicate that V cations exhibit +4/+5 oxidation states, respectively (Supplementary Table 2), which is consistent with the results of XPS spectra (Supplementary Fig. 6).

**Fig. 2:**
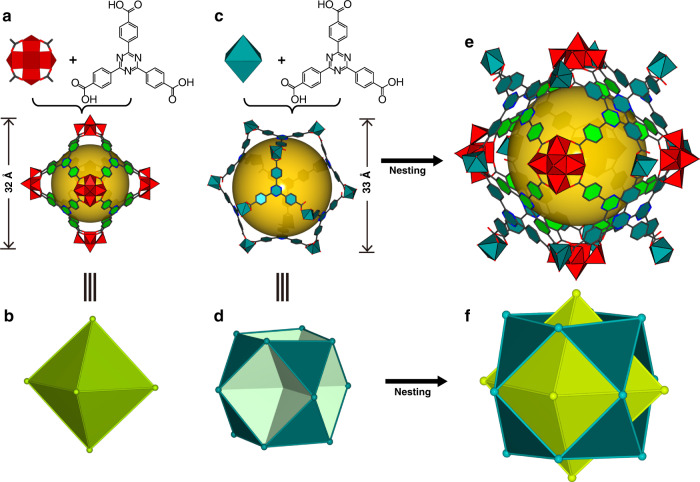
The double-shell structure of 1. **a** The structure of the inner cage constructed by {V_5_O_9_Cl} clusters and TATB ligands. The yellow ball placed in the structure indicates the interior space of the cage. **b** The polyhedral model of the inner cage is represented by an octahedron, in which {V_5_O_9_Cl} clusters are chosen as vertices of the polyhedron and TATB ligands act as faces of a trigon. **c** The structure of the outer cage constructed by V atoms and TATB ligands. **d** The polyhedral model of the outer cage is represented by a cuboctahedron, in which V atoms are chosen as vertices of the polyhedron and TATB ligands act as faces of a trigon. **e** The real nested structure of the inner and outer cages. **f** Schematic representation of the nesting between Archimedean and Platonic polyhedra.

The inner cage is a polyoxovanadate–based metal–organic octahedron, [V_5_O_9_Cl)_6_(TATB)_8_]^12−^, constructed from {V_5_O_9_Cl} clusters and TATB ligands (Fig. 2a, Supplementary Fig. 7a). The {V_5_O_9_Cl} cluster, as a concave secondary building block, consists of an apical vanadium atom (+5) and four basal plane vanadium atoms (+4) bridged by four *μ*_3_–O atoms (Supplementary Fig. 7b) with the V**−**O bond lengths ranging from 1.577 (8) to 2.021 (5) Å. Moreover, the IR spectrum of the double-shell cage shows the characteristic V=O band in the range of 950–990 cm^−1^ (Supplementary Fig. 3). Each {V_5_O_9_Cl} cluster is coordinated with four carboxylate ligands, generating a bowl-shaped motif (Supplementary Fig. 8). Six such concave units are further bridged by eight TATB ligands, thereby affording an octahedron that belongs to one of Platonic solids (Fig. 2b) with the face symbol [3^8^]. The TATB ligands act as the triangular faces, and the {V_5_O_9_Cl} clusters act as the vertices of the octahedron. The outer diameter of the octahedron is approximately 3.2 nm as measured by the longest distance between the outermost oxygen atoms of antipodal vertices. To indicate the interior space, a yellow ball is placed in the cavity^32^ by Diamond program^33^ (Fig. 2a), whose diameter is of ca. 1.9 nm based on the distance between the antipodal Cl ions.

The outer cage, [V_12_O_12_(TATB)_8_(HSO_4_)_12_]^12−^, is constructed by eight TATB ligands and twelve vanadium atoms (Fig. 2c), whose topology is best described as a cuboctahedron, one of Archimedean solids with *F*  = 14, *E* = 24, *V* = 12 (*F*: Face, *E*: Edge, *V*: vertex) (Fig. 2d). The V^...^V distances along the edges of the polyhedron are in the range of 16.376(6)–16.677(6) Å. The largest cross-sectional diameter of this cuboctahedron is about 3.3 nm (metal to metal), and the inner diameter represented by the yellow sphere (Fig. 2c) is of ca. 2.6 nm based on the distance between the antipodal triazine rings. It is noted that the coordination environment of the outer V ions is different from that of the inner ones. Each V^IV^ center is coordinated by two TATB ligands and a sulfite, with the V−O bond lengths ranging from 1.60(2) to 2.431(11) Å.

**Fig. 3:**
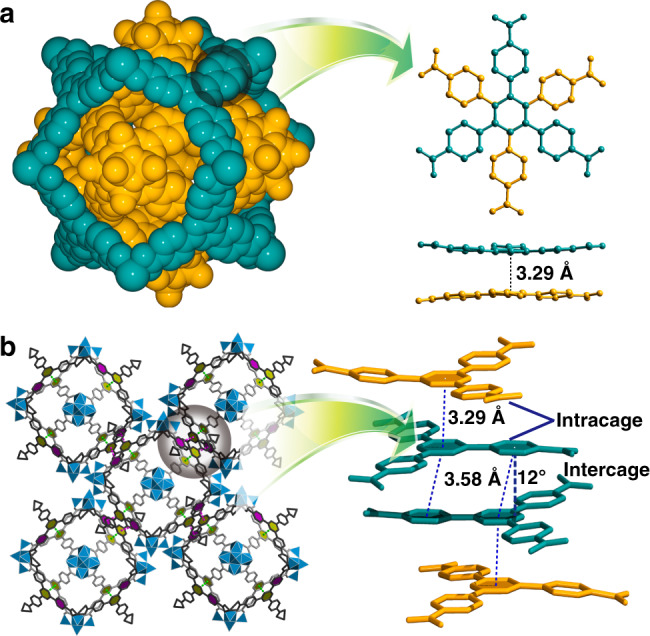
Supramolecular interactions of 1. **a** A face-to-face π–π interaction between the inner and outer cages, where most of the ring–plane area overlaps. Atoms of the inner and outer cages are marked in orange and cyan, respectively. **b** A π–π interaction between the neighboring double-shell cages, where the rings are parallel displaced.

In view of the fact that the inner and outer cages are very close in size, it seems nearly impossible to generate a Matryoshka–type structure at first glance. Is there any specific factor that makes such a case occur? A deeper probe into the geometric relationship of regular polyhedra might be able to give us a lead. As we know, Archimedean polyhedra are derived from Platonic polyhedra by truncated operation. Consequently, a cuboctahedron is created by cutting away six corners of an octahedron, while preserving their own symmetry (both *O*_*h*_ point–group symmetry). Following the above operation, in the current situation, if we restore the cuboctahedron into its precursor polyhedron, a larger octahedron with a diameter of 4.7 nm can be obtained that is sufficiently large to accommodate an octahedron of 3.2 nm. As a result, it is theoretically possible for two polyhedra with approximate size to be nested. The following question is what kind of arrangement the two cages take, that is to say, how do they interact with each other to achieve the ultimate Matryoshka–type architecture? To generate nesting, one feasible strategy is that the six vertices of the inner octahedron thread out from the vacant square polygons of the outer cuboctahedron, as shown in Fig. 2e. Such an intelligent arrangement fashion results in the fact that the TATB ligands of the inner octahedron are nearly parallel with TATB ligands of the outer cuboctahedron, the dihedral angle being 0.972(1)° (Figs. 2f, 3a). Because most of the ring plane areas overlap each other, the face-to-face distance between two triazine rings is of 3.292(2) Å and the centroid–centroid distance is of 3.306(2) Å (Fig. 3a). These values show that there exists a favorable face-to-face π-stacking within the double-shell cage. This face-to-face stacked orientation, as evidenced by the survey based on a Cambridge Structural Database search, usually gives rise to the value of centroid–centroid contacts slightly below 3.4 Å^34–36^. Under the guidance of the supramolecular recognition, a double-shell superstructure, composed of nested Archimedean and Platonic polyhedra, is ultimately formed (Fig. 2f). We thus speculate that this type of strong π–π interactions promote the formation of the hierarchically organized structure as the main driving force. After nesting, the surface of **1** is almost fully enclosed (Fig. 3a) as confirmed by the N_2_ sorption measurements (Supplementary Fig. 9). Yet, the two triazine rings from the neighboring double-shell cages are parallel displaced with respect to each other.

The centroid–centroid contact is of 3.577(2) Å, and the displacement angle is 12° that is measured by the ring–centroid vector and the ring normal to one of the triazine planes (Fig. 2b). To obtain quantitative insight into the energetics of the π–π stacking interactions, we performed theoretical calculations. The relative energy of two model structures (**D1** and **D2**) was evaluated at the M062x/6–311++G(d,p) level (see the Method for computational details). As shown in Supplementary Fig. 10, **D1** represents the interaction fragment between the inner and outer cages in the double-shell cage, which has the aromatic π–π stacking interactions caused by the nearly overlapping s-triazine molecules. Whereas **D2** represents the fragment between the double-shell cages, which has a head-to-tail phenyl-s-triazine overlap region. The calculation results show that **D1** is more stable than **D2** by 5.0 kcal mol^–1^. Note that there are eight above-mentioned fragments in the complete double-shell cage, it can be expected that the aromatic π–π stacking interactions between inner and outer cages become much stronger than those between adjacent double-shell cages. In addition, Supplementary Fig. 11 shows the frontier molecular orbital distribution for **D1** and **D2**. More extended conjugation obviously contributes to the formation of such a double-shell structure.

Given that the inner and outer cages in **1** are independent and assembled by supramolecular interactions, we try to obtain their discrete objects. On the basis of our research experience about polyoxovanadate-based MOPs^37,38^, the inner cage, (NH_2_Me_2_)_12_[(V_5_O_9_Cl)_6_(TATB)_8_]∙(CH_3_OH)_4_ (**1a**), was successfully isolated in high yield by reaction of VCl_4_, VOSO_4_, and H_3_TATB in DMF:CH_3_CN:CH_3_OH (4:1:1 v/v) at 130 °C for 48 h (see the “Methods” section for a detailed synthesis procedure). Singe-crystal diffraction revealed that **1a** crystallized in cubic system with space group *Fm–3m* (Supplementary Table 1). Nevertheless, no matter how we change the reaction conditions, the outer cage cannot be isolated from the raw materials. We thus speculate that the inner cage **1a** might induce the formation of the outer cage through template effects. To further investigate the assembly mechanism, the prefabricated **1a** was directly added to the reaction system containing VOSO_4_ and H_3_TATB in DMF:CH_3_CN (4:1, v/v) solution at 130 °C. As expected, the double-shell structure was successfully obtained. This result suggests that the inner cage might serve as an anion template in the self-assembly, and eventually leads to the formation of the double-shell architecture.

### Selective adsorption for dye molecules

**Fig. 4:**
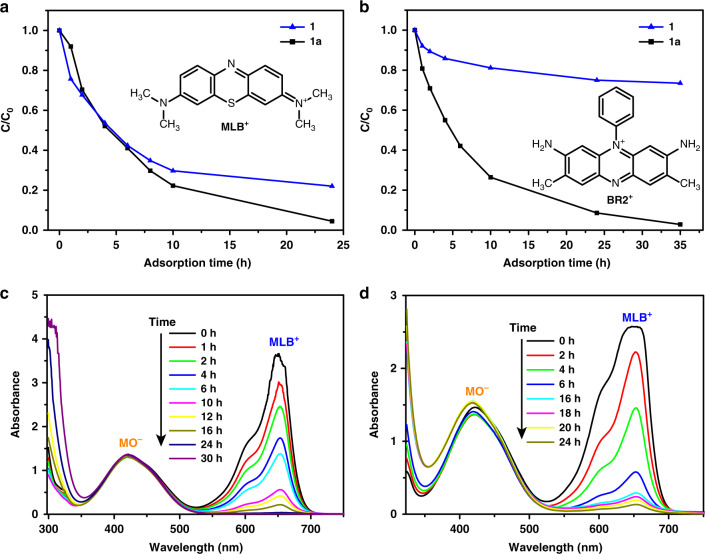
Selective adsorption of 1 and 1a for dye molecules. **a**, **b** Concentration changes of cationic dyes MLB^+^ and BR2^+^ with time as determined by the UV–Vis absorbance at a selected wavelength. **c**, **d** Temporal evolution of UV–Vis absorption spectra of a MLB^+^/MO^−^ mixture in the presence of **1** and **1a**.

As a result of the high negative charge and the good stability of **1** and **1a**, they are anticipated to adsorb polycyclic molecules in solution. Three dye molecules with different sizes and charges were selected: Methylene Blue (MLB^+^, 4.00 × 7.93 × 16.34 Å), Basic Red 2 (BR2^+^, 6.43 × 11.34 × 13.43 Å), and Methyl Orange (MO^−^, 5.31 × 7.25 × 17.39 Å). Fresh crystals of **1** or **1a** (15.0 mg) were immersed in 10 mL ethanol solution of dyes (concentration: [MLB^+^] = 5 × 10^–5^ M, [BR2^+^] = 2 × 10^–4^ M, [MO^−^] = 1 × 10^–4^ M, respectively). The amounts of dye molecules in the supernatant was monitored by UV–Vis spectrophotometry (characteristic absorbance: MLB^+^ 653 nm, BR2^+^ 545 nm, MO^−^ 421 nm). As shown in Supplementary Fig. 12, the concentrations of MLB^+^ and BR2^+^ in ethanol solution decreased gradually with time, but the concentration of MO^−^ remained unchanged. These results preliminarily show that only cationic dye molecules can be selectively absorbed by **1** or **1a**. It is noteworthy that although **1** and **1a** show similar adsorption abilities to MLB^+^ (Fig. 4a), the adsorption ability of **1** for the larger BR2^+^ molecule is obviously lower than that of **1a**, (Fig. 4b), suggesting that **1** is sensitive to the size of dye molecules. The inherent reason for this difference can be explained from the packing architectures of **1** and **1a**. As shown in Supplementary Fig. 13, there are one-dimensional intersecting channels in the packing diagram of **1a** with a diameter of about 8 Å, which can easily accommodate MLB^+^ or BR2^+^ molecules. Nevertheless, there are no obvious pores in the packing model of **1** due to the close π–π interactions between adjacent molecules; therefore only the small-sized cationic MLB^+^ dyes could be effectively adsorbed by **1**. To further demonstrate the selective adsorption of **1** and **1a** for cationic dye molecules, the crystalline samples were soaked into a DMF solution of MLB^+^ and MO^−^ dye molecules. UV–Vis spectra distinctly showed that only the cationic MLB^+^ was adsorbed by **1** and **1a** (Fig. 4c, d). Thus, it can be concluded that an ion–exchange process occurred between the cationic dyes and [NH_2_Me_2_]^+^ cations^37^.

### Selective encapsulation for guest molecules of 1a

**Fig. 5:**
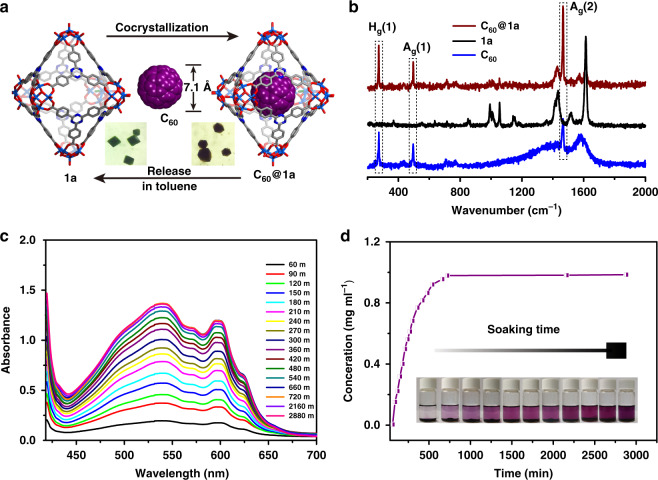
The inclusion and release of C_60_ molecules in 1a. **a** A schematic illustration of the inclusion and release of C_60_ molecules in **1a**. **b** Raman spectra of C_60_, **1a** and **C**_60_**@1a**. **c** Temporal evolution of UV–Vis absorption spectra of C_60_ in toluene solution. **d** Concentration changes of C_60_ in toluene solution with time. The inset figure shows the color evolution of the toluene solution with time.

Owing to the high yield and the large void of the independent inner cage, **1a** is highly promising as a molecular receptor for encapsulation of various guests. We first tried to include the colored organic molecule C_60_ in **1a**. The failure of direct adsorption by placing **1a** into the C_60_–toluene solution turned our attention to in situ co-crystallization. We added C_60_ (~7.1 Å) into the synthesis system of **1a**. After co-crystallization for two days, the crystal color, as shown in Fig. 5a, underwent a visible change from green to dark brown, indicating the successful encapsulation of C_60_ molecule into the void of **1a**. The inclusion of C_60_ in **1a** was also probed by Raman spectroscopy with 488 nm excitation (Fig. 5b). The peaks of H_g_(1) 274 nm, A_g_(1) 497 nm, A_g_(2) 1465 nm, and H_g_(8) 1576 nm are attributed to the vibrations of C_60_, in which the A_g_(2) vibration is the characteristic peak of C_60_^39^. What is particularly remarkable is we have got the crystal data of C_60_@**1a** which provide direct evidence for C_60_ encapsulation (Supplementary Table 1). X-ray structural analysis clearly shows that one molecule of **1a** is capable of encapsulating one C_60_ molecule, which is located in the center of the cavity (Fig. 5a, Supplementary Fig. 14a). Careful examination identifies that the triazine rings of the cage surface are parallel with the six-membered rings of C_60_, but the centroid–centroid distance of 5.960(2) Å exceeds 3.80 Å, the maximum acceptable contact for π–π interactions (Supplementary Fig. 14b). We therefore infer the binding of C_60_ occurs through van der Waals forces. Meanwhile, the capability of C_60_**@1a** to release C_60_ in toluene has also been tested. Fresh crystals C_60_@**1a** were soaked in the toluene solution, and the amount of C_60_ molecules in the toluene was measured by UV–Vis spectrophotometry (characteristic absorbance: 540 nm and 600 nm). As shown in Fig. 5c, the plots of the C_60_ concentrations versus time clearly showed a continuous increase which is indicative of the gradual release of C_60_ molecules, and eventually the release amount reached equilibrium after 12 h. The subsequent quantitative analyses based on the values at 540 nm revealed that 50 mg of C_60_@**1a** can release 4.8 mg of C_60_, which is consistent with the expected ratio (the calculated mass fraction of C_60_ in C_60_@**1a** is 1:10). At the end of the release experiments, the toluene solution changed from colorless to violet (Fig. 5d), and the crystals turned green again but were still crystalline (Fig. 5a).

Inspired by the successful encapsulation of C_60_ molecule, we used another two π-conjugated molecules (Supplementary Fig. 15), anthracene (flaky structure, 7.3 × 2.8 Å) and C_70_ (spherical structure, 8.1 Å), to further investigate the encapsulation capacity of **1a** for various guest species, so as to gain some insight into the inclusion chemistry of **1a**. The co-crystallization of anthracene with VOSO_4_, VCl_4_ and H_3_TATB yielded green product. The emission spectrum of the green product in solid-state showed the characteristic emission peaks of anthracene in the range of 400–600 nm with a slight blue shift (Supplementary Fig. 16), implying anthracene molecule might be trapped within **1a**. The exact location of anthracene molecule was further confirmed by X-ray crystallography. Unlike the C_60_ molecule, anthracene molecule was located in the interstitial space between adjacent cages (Supplementary Fig. 17). This is not surprising considering that the diameter of anthracene is comparable to that of C_60_. However, attempts to in situ encapsulate C_70_ molecule turned out to be failures as demonstrated by the product color (green) and Raman spectroscopy (Supplementary Fig. 18). We attribute the failure to the relatively large size of C_70_. Except for the molecular size, the guest concentration may also be another factor for the binding process. The order of the solubility of these three guest molecules in the reaction system is anthracene > C_60_ > C_70_. As the solubility of C_60_ is superior to that of C_70_, the concentration of C_60_ in the reaction system is inevitably higher than that of C_70_, which might be a favorable dynamic factor for the formation of C_60_@**1a**.

### Magnetic properties

**Fig. 6:**
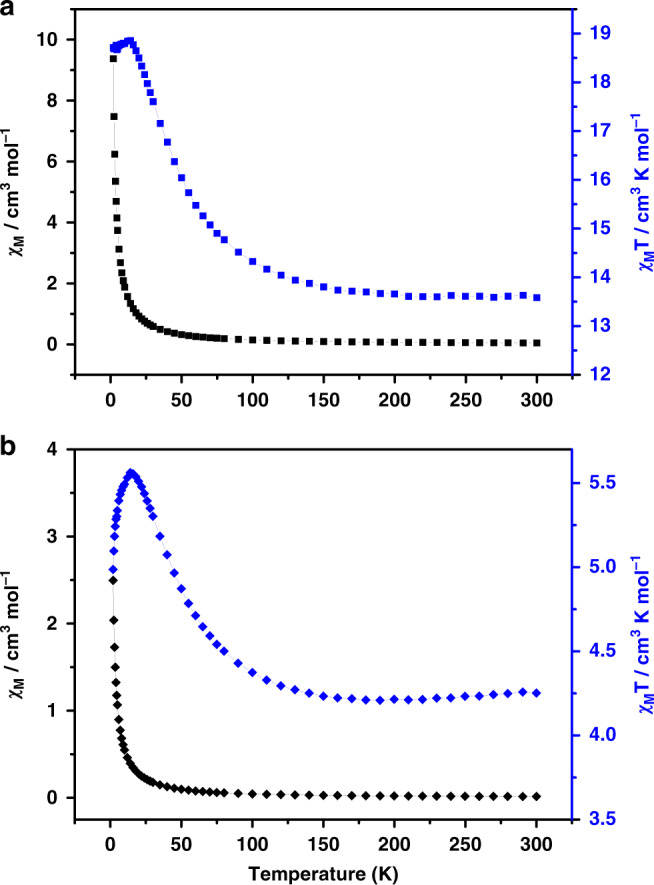
Magnetic data for 1 and 1a. Temperature dependence of χ_M_ and χ_M_*T* versus *T* plots for **1** (**a**) and **1a** (**b**).

In view of the fact that the inner cage **1a** can be isolated, the magnetic properties of **1** and **1a** were explored to study the subtle magnetic differences originated from structure. The variable temperature magnetic susceptibilities were measured by using the fresh crystalline samples of **1** and **1a** from 2 to 300 K under a magnetic field of 1000 Oe (Fig. 6). For **1**, the room temperature χ_M_*T* value is 13.6 cm^3^ K mol^–1^, which is consistent with the excepted value of 13.5 cm^3^ K mol^–1^. Whereas for **1a**, the χ_M_*T* value of 4.2 cm^3^ K mol^–1^ is much lower than the excepted value (9.0 cm^3^ K mol^–1^). The magnetism is contributed by the tetravalent V^IV^ ions rooting in outer-shell uninucleate V^IV^ and {V_5_O_9_Cl} clusters, wherein the {V_5_O_9_Cl} includes four uncoupled V^IV^ (*S* = 1/2, *g* = 2.00)^19,40,41^ and a central diamagnetic V^V^ ion. As the temperature decreased from 300 to 100 K, the changes of χ_M_*T* vs. *T* data for **1** and **1a** trend to be similar, namely the χ_M_*T* value of both **1** and **1a** slightly decrease. Further lowering the temperature, the value increases rapidly reaching the maximum of 18.8 cm^3^ K mol^–1^ and 5.6 cm^3^ K mol^–1^ for **1** and **1a**, respectively, at 14 K. Subsequently for **1**, the value slightly drops to 18.6 cm^3^ K mol^–1^ at 2 K. But the χ_M_*T* value of **1a** decreases sharply to 4.9 cm^3^ K mol^–1^ at 2 K. The continuous increase with reduction of temperature at high temperatures indicates intramolecular ferromagnetic interactions between the neighboring V^IV^ ions (*S* = 1/2, *g* = 2.00). The plot of 1/χ_M_ versus *T* can be fitted with the Curie−Weiss law in the temperature range from 2 to 300 K and two positive Weiss constants *θ* = 4.61 and 3.70 K for **1** and **1a** were respectively obtained (Supplementary Figs. 19–20). Both the curve and two positive *θ* further indicate the weak ferromagnetic coupling interactions within the double-shell cage or the independent interior cage.

## Discussion

As mentioned in the beginning, it was believed to be challenging to create nanoscale hierarchical structures. Here we not only have succeeded in assembling a fascinating cage-within-cage molecule but also several distinctive nesting characteristics have been brought to light in this case. First, a new way of using supramolecular recognition for the interconnection between inside and outside cages is demonstrated; this finding might provide a rational basis for assembling non-natural shell-like structures at the molecular level. Second, nesting first occurs in cages almost with the same size, breaking through the traditional restraints of appreciable “small vs. large” pattern. Third, the inside cage of the double-shell can be isolated as a discrete cage. Moreover, initial inclusion studies for guest molecules suggest that the inner cage can act as a molecular receptor to selectively trap the guests. We believe these out-of-ordinary nesting traits and the nesting strategy illustrated here establish new guidelines for the construction of Matryoshka–type superstructures, and we expect more such examples can be artificially synthesized in the near future.

## Methods

### Materials and characterization

All the reagents were obtained from commercial sources and used without further purification. PXRD measurements were recorded ranging from 5 to 50º at room temperature on a Siemens D5005 diffractometer with Cu–*Kα* (*λ* = 1.5418 Å). The C, H and N elemental analysis were conducted on a Perkin–Elmer 2400CHN elemental analyzer. TGA of the samples was performed using a Perkin–Elmer TG–7 analyzer heated from room temperature to 800 ºC under nitrogen at the heating rate of 10 °C min^–1^. IR spectrum was performed in the range of 4000–400 cm^–1^ using KBr pellets on an Alpha Centaurt FT/IR spectrophotometer. The N_2_ sorption tests were measured on automatic volumetric adsorption equipment (Belsorp mini II). Excitation and emission spectra were obtained on a SPEX FL–2T2 spectrofluorometer equipped with a 450 W xenon lamp as the excitation source. Scanning electron microscopy (SEM) images were carried out using an XL–30 ESEM–FEG scanning electron microscope. XPS was performed using an Escalab 250 instrument. The UV–visible absorption spectra were obtained with a Shimadzu UV–2550 spectrophotometer. Variable temperature magnetic susceptibility data were obtained in the temperature range 2–300 K using a SQUID magnetometer (Quantum Design, MPMS–5) with an applied field of 1000 Oe.

### Synthesis of (NH_2_Me_2_)_24_{[(V_5_O_9_Cl)_6_(TATB)_8_][V_12_O_12_(TATB)_8_(HSO_4_)_12_]}∙(CH_3_OH)_16_ (1)

H_3_TATB (25.0 mg, 0.057 mmol), VOSO_4_ (25 mg, 0.15 mmol), VCl_3_ (20 mg, 0.13 mmol), and NaVO_3_ (18 mg, 0.1 mmol) were dissolved in DMF (2 mL), CH_3_OH (0.5 mL), and CH_3_CN (0.5 mL). Then the mixture was sealed in a Parr Teflon-lined stainless steel vessel and heated at 130 °C for two days and gradually cooled to room temperature. Dark green crystals were obtained, washed with DMF, and dried in air (yield: ~43%, based on H_3_TATB). Elemental analysis (%) for C_448_H_460_Cl_6_N_72_O_226_S_12_V_42_: Calcd: C, 40.75; H, 3.51; N, 7.63. Found: C, 40.58; H, 3.75; N, 7.32. IR (KBr, cm^–1^): 3424(s), 1708(s), 1582(m), 1518(m), 1465(w), 1402(m), 1360(s), 1111(s), 974(s), 883(w), 827(m), 770(s), 695(w), 619(s), 532(s).

### Synthesis of (NH_2_Me_2_)_12_[(V_5_O_9_Cl)_6_(TATB)_8_]∙(CH_3_OH)_4_ (1a)

H_3_TATB (25.0  mg, 0.057 mmol), VOSO_4_ (25 mg, 0.15mmol), and VCl_4_ (20 mg, 0.104 mmol) were dissolved in DMF (2 mL), CH_3_OH (0.5 mL), and CH_3_CN (0.5 mL). Then the mixture was placed in a Teflon–lined stainless vessel, and heated to 130 °C for two days. After slowly cooling to room temperature, green crystals were obtained, washed with DMF and dried in air (yield: ~69%, based on H_3_TATB). Elemental analysis (%) for C_220_H_208_Cl_6_N_36_O_106_V_30_: Calcd: C, 38.89; H, 3.08; N, 7.42. Found: C, 38.78; H, 3.25; N, 7.27.IR (KBr, cm^–1^): 3410(s), 1713(s), 1556(m), 1519(m), 1483(w), 1403(m), 1359(s), 1257(w), 1103(w), 987(s), 881(s), 824(s), 771(m), 698(s), 588(w), 551(w). **1a** can be obtained in a wide range of temperatures. As the reaction temperature is decreased to 90 °C, **1a** still can be obtained, but the yield drops significantly.

### Synthesis of 1 from 1a

In a 15 mL Teflon-lined stainless vessel, prefabricated crystals of **1a** (10 mg), VOSO_4_ (20 mg), and H_3_TATB (20 mg) were dissolved in DMF (2 mL) and CH_3_CN (0.5 mL). The mixture was heated at 130 °C for 48 h and gradually cooled to room temperature to obtain dark green crystals of **1** (yield: ~52%, based on H_3_TATB).

### Synthesis of C_60_@1a

The procedure is similar to that of **1a**, except that extra 5 mg C_60_ and 1 mL toluene was added into the reaction system. After cooling to the room temperature, dark brown crystals were obtained and washed with toluene twice to give the pure samples (yield: ~53%, based on H_3_TATB). Elemental analysis (%) for C_280_H_208_Cl_6_N_36_O_106_V_30_, Calcd: C, 44.75; H, 2.79; N, 6.71. Found: C, 44.54; H, 2.64; N, 6.57.

### Procedure for trying to encapsulate C_70_ in 1a

The procedure is similar to the methods mentioned above for the synthesis of C_60_**@1a**, except that equal quality of C_70_ instead of C_60_. Unfortunately, after cooling to the room temperature, just the green crystals of **1a** were obtained. We also tried to change the amount of C_70_ but still got the same results.

### Synthesis of anthracene@1a

The procedure is similar to that of **1a**, except that extra 15 mg anthracene was added into the reaction system. After cooling to the room temperature, green crystals were obtained, washed with DMF twice to give the pure samples (yield: ~60%, based on H_3_TATB).

### C_60_ extraction from C_60_@1a

Inclusion crystals C_60_@**1a** were immersed into 5.0 mL of toluene (spectroscopic grade). The resulting suspension was stand at room temperature without crashing crystals. The concentration of the extracted C_60_ was measured by UV–Vis absorption spectra of the supernatant (detection wavelength: 540 nm).

### Single-crystal X-ray diffraction

A summary of the crystallographic data and structural refinements for **1**, **1a** and C_60_@**1a** and anthracene@**1a** are given in Supplementary Table 1. All crystallographic data were collected at 173 K on a Bruker D8–Venture diffractometer with graphite–monochromated Mo K*α* radiation (*λ* = 0.71073 Å) (**1a**) and Cu K*α* radiation (*λ* = 1.5418 Å) (**1**, C_60_@**1a** and anthracene@**1a**). The data were collected using the program *APEX 3* and processed using the program SAINT routine in *APEX 3*. The structures were solved by direct methods with SHELXS–2014 and refined with SHELXL–2014 program^42,43^. All non–hydrogen atoms, except disordered C_60_ and anthracene, were refined in anisotropic approximation. Hydrogen atoms were refined in geometrically calculated positions using the “riding model” with *U*_*iso*_*(H)* = 1.2*U*_*iso*_*(C)*. The large cell volume and high crystal symmetry did not allow refining the disordered solvent molecules and dimethylamine counter cations within the crystal lattices, therefore, SQUEEZE routines in PLATON were used for **1**, **1a**, and C_60_@**1a** to generate the reflection intensities with subtracted solvent contributions^44^. For **1**, the sulfate groups were disordered, so the restraints DFIX and SADI were used to confine the bond length of sulfate groups. SIMU and ISOR constraints were used for organic ligands and partial metal centers with large thermal motions. For C_60_@**1a**, the C_60_ molecule was disordered, so the restraints SADI, FLAT, SIMU, RIGU, ISOR were applied to model the geometry of C_60_, the ISOR constraints were used for dimethylamine cation with large thermal motion. For anthracene@**1a**, the anthracene and dimethylamine molecule were disordered, so the restraints SADI, FLAT, RIGU, and SIMU were used to confine the geometry of anthracene molecule, DFIX, ISOR, and SIMU constraints were used for dimethylamine cation with large thermal motion.

### Computational details

Two model structures **D1** and **D2** were extracted from crystal structures. Constraint optimization was performed at the M062x^45^/6–31G(d) level to determine the position of hydrogen atoms. Electronic energies were evaluated at the M062x/6–311++G(d,p) level. These calculations were carried out with the Gaussian 09 program^46^.

## Supplementary information

Supplementary Information

## Data Availability

The X-ray crystallographic data for structures reported in this article have been deposited at the Cambridge Crystallographic Data Centre (CCDC), under deposition number CCDC 1864118 (**1**), 1864121 (**1a**), 1864122 (C_60_@**1a**), 2003560 (anthracene@**1a**). These data can be obtained free of charge from The Cambridge Crystallographic Data Centre via www.ccdc.cam.ac.uk/data_request/cif. All relevant data supporting the findings of this study are available from the corresponding authors on request.
