# Supplementary material for: Equi–size nesting of Platonic and Archimedean metal–organic polyhedra into a twin capsid
Source: Nat Commun. 2020 Aug 14;11:4103. doi: 10.1038/s41467-020-17989-6 (PMC7429837; doi:10.1038/s41467-020-17989-6)
Supplement: Supplementary file 1 — Supplementary Information [file 41467_2020_17989_MOESM1_ESM.pdf]

Supplementary Information for

Equi–size nesting of Platonic and Archimedean metal–organic polyhedra into a twin capsid

Gan *et al*

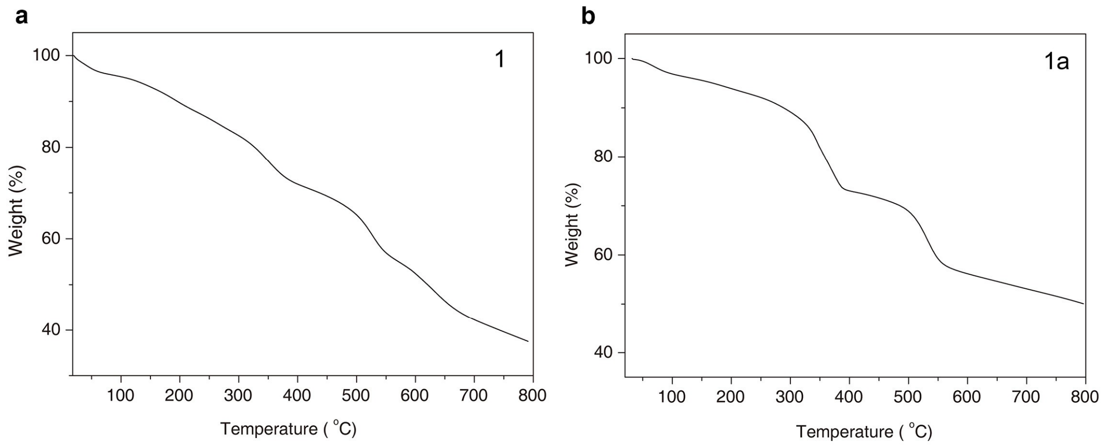

Supplementary Fig. 1. TG curves for **1** (a) and **1a** (b).

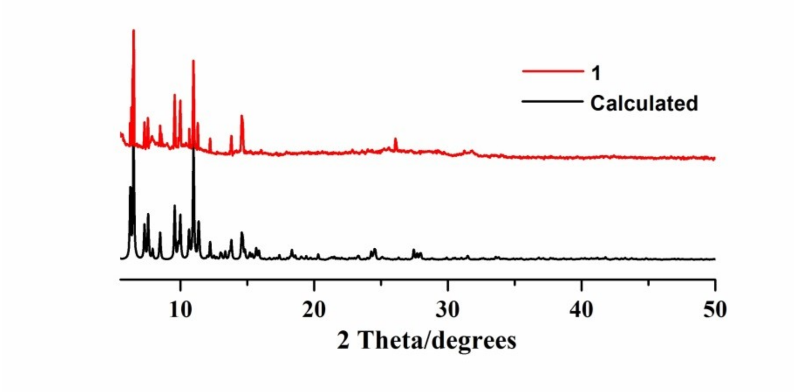

Supplementary Fig. 2. Calculated (black) and observed (red) PXRD patterns for **1**.

Supplementary Table 1. The crystallographic data for 1, 1a, C_{60}@1a and anthracene@1a

Identification code	**1**	**1a**	**C_{60}@1a**	**anthracene@1a**
Empirical formula	$C_{448}H_{460}Cl_6N_{72}O_{226}S_{12}V_{42}$	$C_{220}H_{208}Cl_6N_{36}O_{106}V_{30}$	$C_{280}H_{208}Cl_6N_{36}O_{106}V_{30}$	$C_{222}H_{154}Cl_6N_{32}O_{102}V_{30}$
Formula weight	13205.75	6793.11	7513.71	6642.66
Temperature/K	173.02	173.02	173.02	173.15
Crystal system	tetragonal	cubic	cubic	cubic
Space group	*I4/m*	*Fm-3m*	*Fm-3m*	*Fm-3m*
a/Å	33.0361(12)	40.0473(9)	39.8949(3)	39.972(7)
b/Å	33.0361(12)	40.0473(9)	39.8949(3)	39.972(7)
c/Å	35.949(3)	40.0473(9)	39.8949(3)	39.972(7)
α/°	90	90	90	90
β/°	90	90	90	90
γ/°	90	90	90	90
Volume/Å^3	39234(4)	64227(4)	63496.9(14)	63867(33)
Z	2	4	4	4
ρ_{calc}g/cm^3	1.118	0.703	0.786	0.691
μ/mm^1	5.076	0.479	4.134	4.065
F(000)	13440.0	13680	15120	13272.0
Radiation	CuKα (λ = 1.54178)	MoKα (λ = 0.71073)	CuKα (λ = 1.54178)	CuKα (λ = 1.54178)
2θ range/°	3.782 to 125.85	4.548 to 50.044	6.266 to 133.446	3.828 to 127.786
Reflections collected	67155	33865	25850	24052
Independent reflections	15660 [R_{int}= 0.0783, R_{sigma}= 0.0860]	2778 [R_{int} = 0.0459, R_{sigma} = 0.0218]	2673 [R_{int} = 0.0464, R_{sigma} = 0.0270]	2624 [R_{int} = 0.0459, R_{sigma} = 0.0283]
Goodness-of-fit on F^2	0.981	1.057	1.092	1.068
Final R indexes [*I*>=2σ (*I*)]	R_1^{a} = 0.1102, wR_2^{b} = 0.2617	R_1^{a} = 0.0440, wR_2^{b} = 0.1651	R_1^{a} = 0.0991, wR_2^{b} = 0.3075	R_1^{a} = 0.0861, wR_2^{b} = 0.2816
Final *R* indexes [all data]	R_1^{a} = 0.1414, wR_2^{b} = 0.2782	R_1^{a} = 0.0547, wR_2^{b} = 0.1783	R_1^{a} = 0.1078, wR_2^{b} = 0.3198	R_1^{a} = 0.0968, wR_2^{b} = 0.2940

[a] $R_1 = \Sigma||F_o|-|F_c||/\Sigma|F_o|$; [b] $wR_2=\{\Sigma [w(F_o^2-F_c^2)^2]/\Sigma [w(F_o^2)^2]\}^{1/2}$

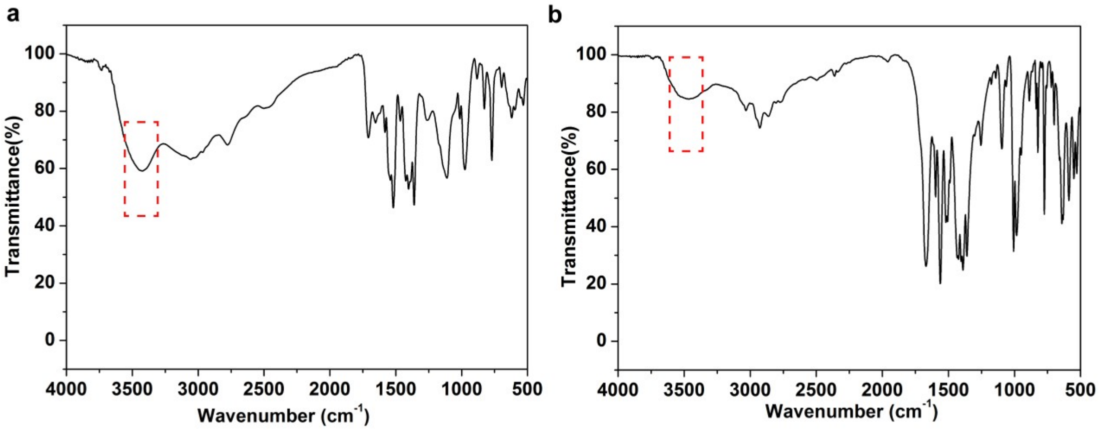

Supplementary Fig. 3. IR spectra of **1** (a) and **1a** (b).

Supplementary Note 1: IR spectra of **1** and **1a** exhibit the characteristic bands at 3500 cm^{-1} (red dashed label), which are corresponding to N-H stretch.

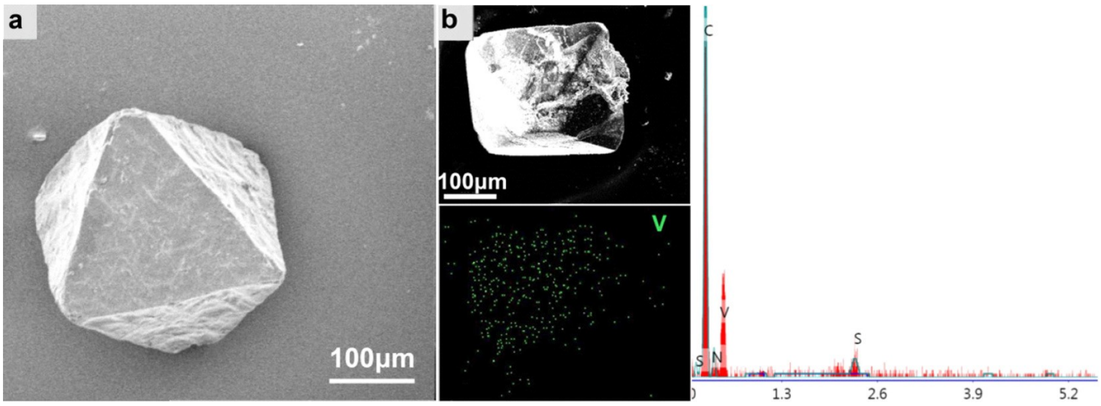

Supplementary Fig. 4. SEM image (a) and EDS mapping (b) of **1**.

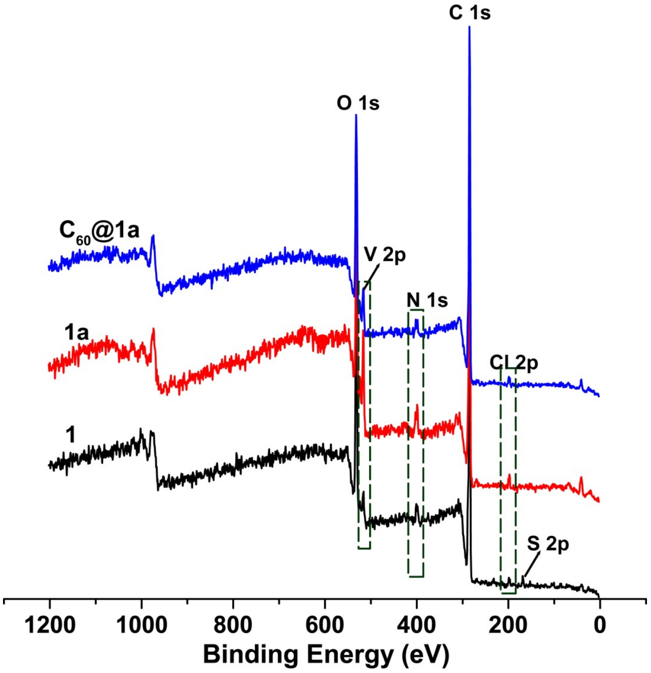

Supplementary Fig. 5. X-ray photoelectron spectroscopy (XPS) of **1**, **1a**, C_{60}@**1a**.

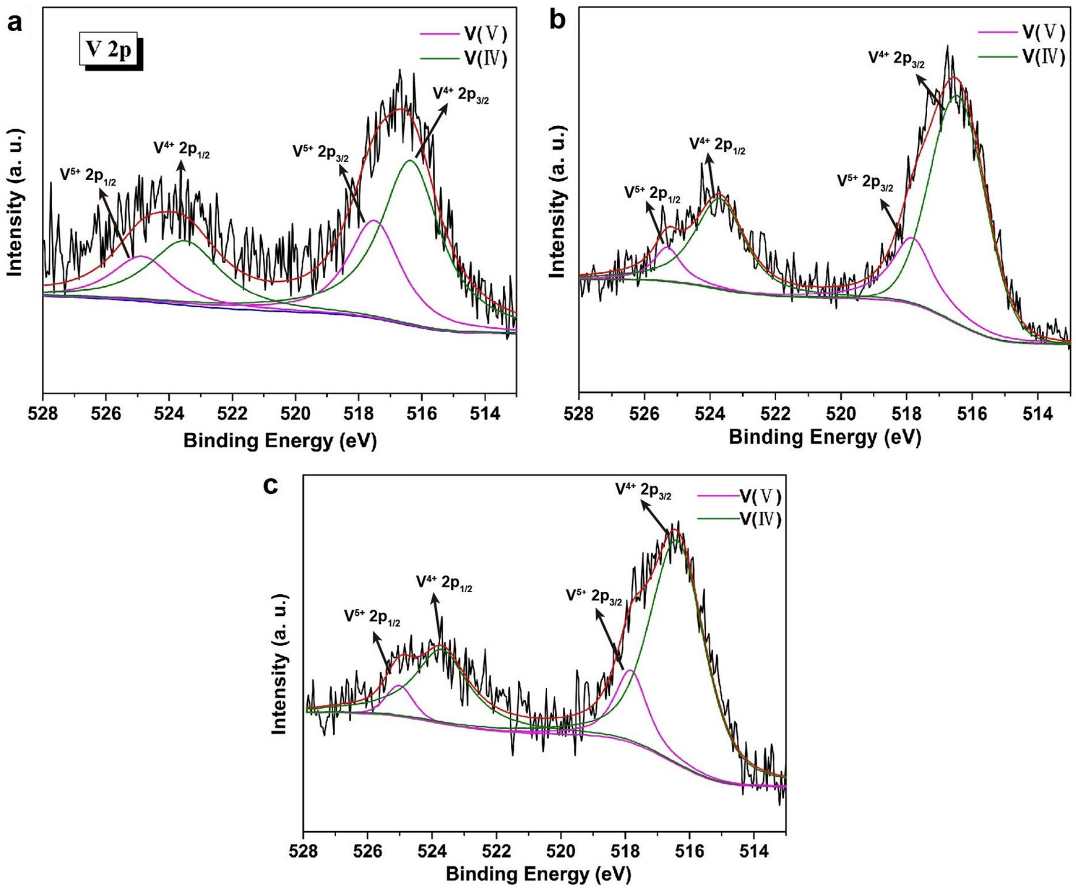

Supplementary Fig. 6. The V 2p XPS spectra of **1** (a), **1a** (b), and C_{60}@**1a** (c).

Supplementary Table 2. BVS results for the vanadium atoms of **1**

	Atom	BVS calc. for V
+5	V4	4.97
	V5	4.81
+4	V1	4.03
	V2	3.91
	V3	4.03
	V6	4.02
	V9	3.93
	V10	4.35

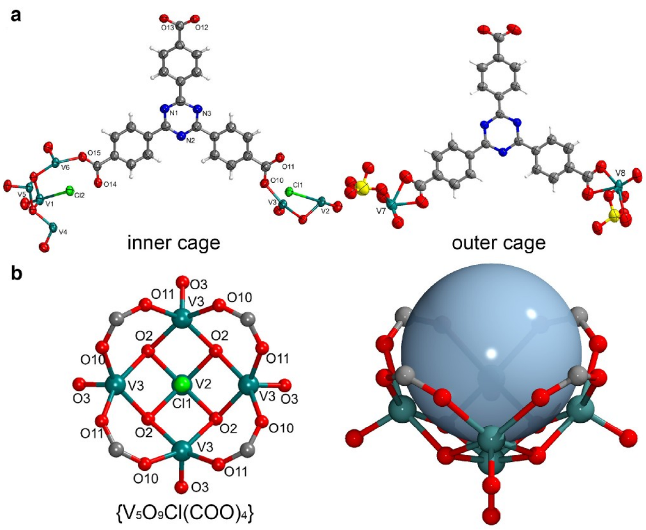

Supplementary Fig. 7. a) .ORTEP diagram of the inner and outer cages. The labeling atoms are shown with thermal ellipsoids at 50% probability. b) Ball-and-stick representation of the $\{V_5O_9Cl(COO)_4\}$ cluster and its concave configuration.

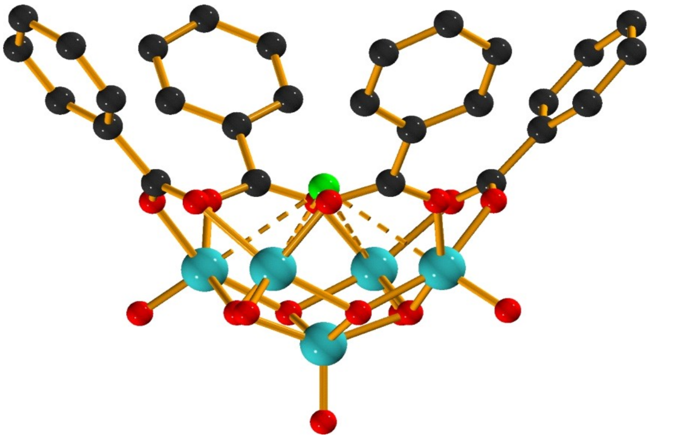

Supplementary Fig. 8. The ball–and–stick view of the bowl-shape motif.

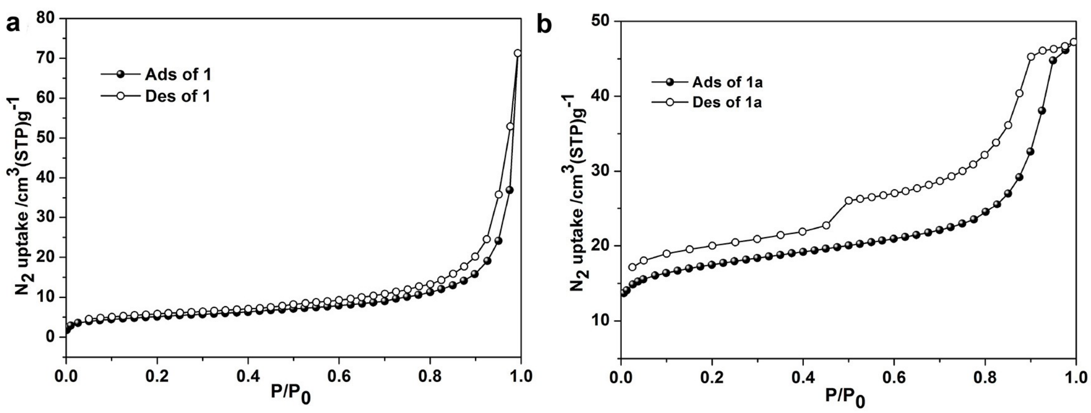

Supplementary Fig. 9. N_2 sorption isotherms at 77 K for **1** (a) and **1a** (b).

Supplementary Note 2: The samples of **1** and **1a** were prepared by exchanging with methanol for three days and then degassing at 60°C for 6 h in a vacuum. N_2 sorption measurements were carried out for the fully activated samples to evaluate the gas accessible porosity. Supplementary Fig. 9 presented N_2 gravimetric uptake at 77 K, which were observed to be 77.4 cm^3 g^{-1} for **1** and 47.3 cm^3 g^{-1} for **1a**. The small uptake is very commen for cages in that their small apertures or windows prohibit gas molecules from accessing the cavity interior.[1-3] For the cases of **1** and **1a**, the windows (~2.3 × 2.4 Å for **1** and ~2.4 × 4.6 Å for **1a**) are too small with respect to N_2 (kinetic diameter 3.64 Å) to allow the passage of N_2.

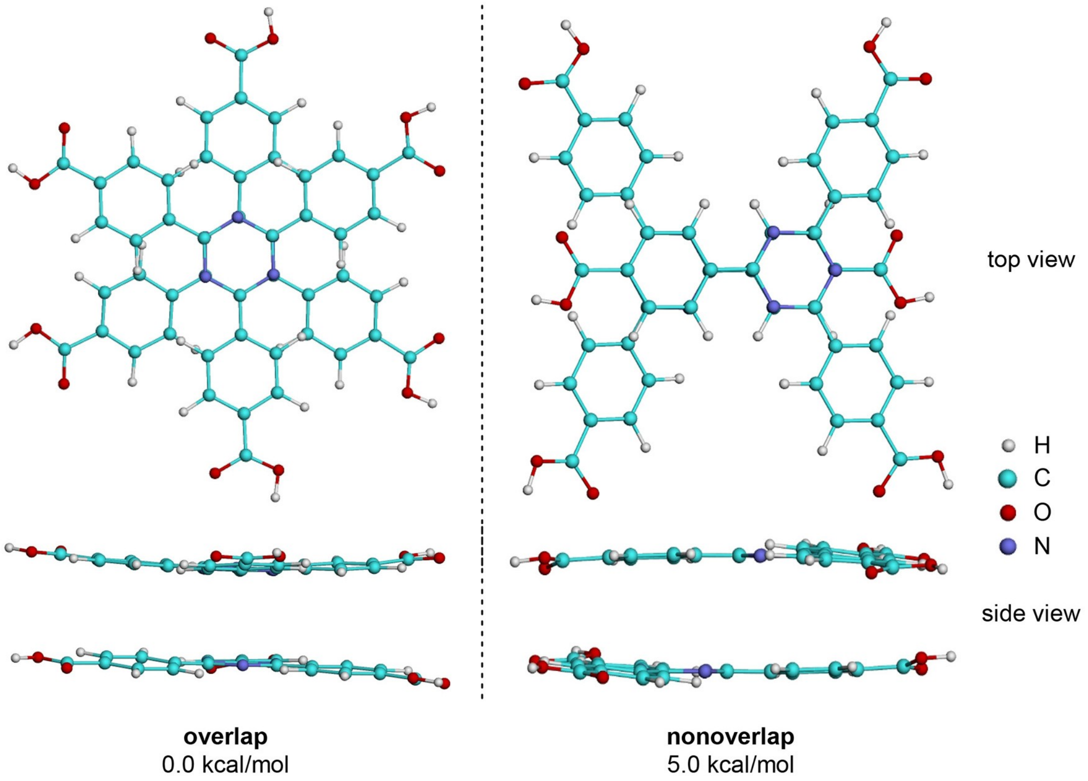

Supplementary Fig. 10. Structures and relative energies (in kcal mol^{-1}) of **D1** and **D2**.

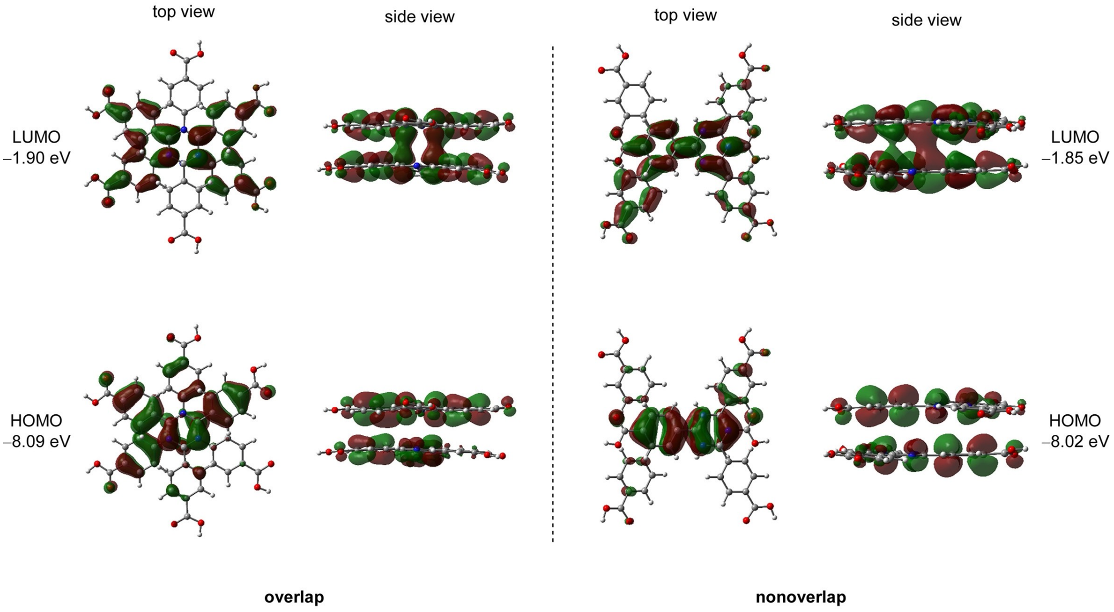

Supplementary Fig. 11. Frontier molecular orbital distribution for **D1** and **D2**.

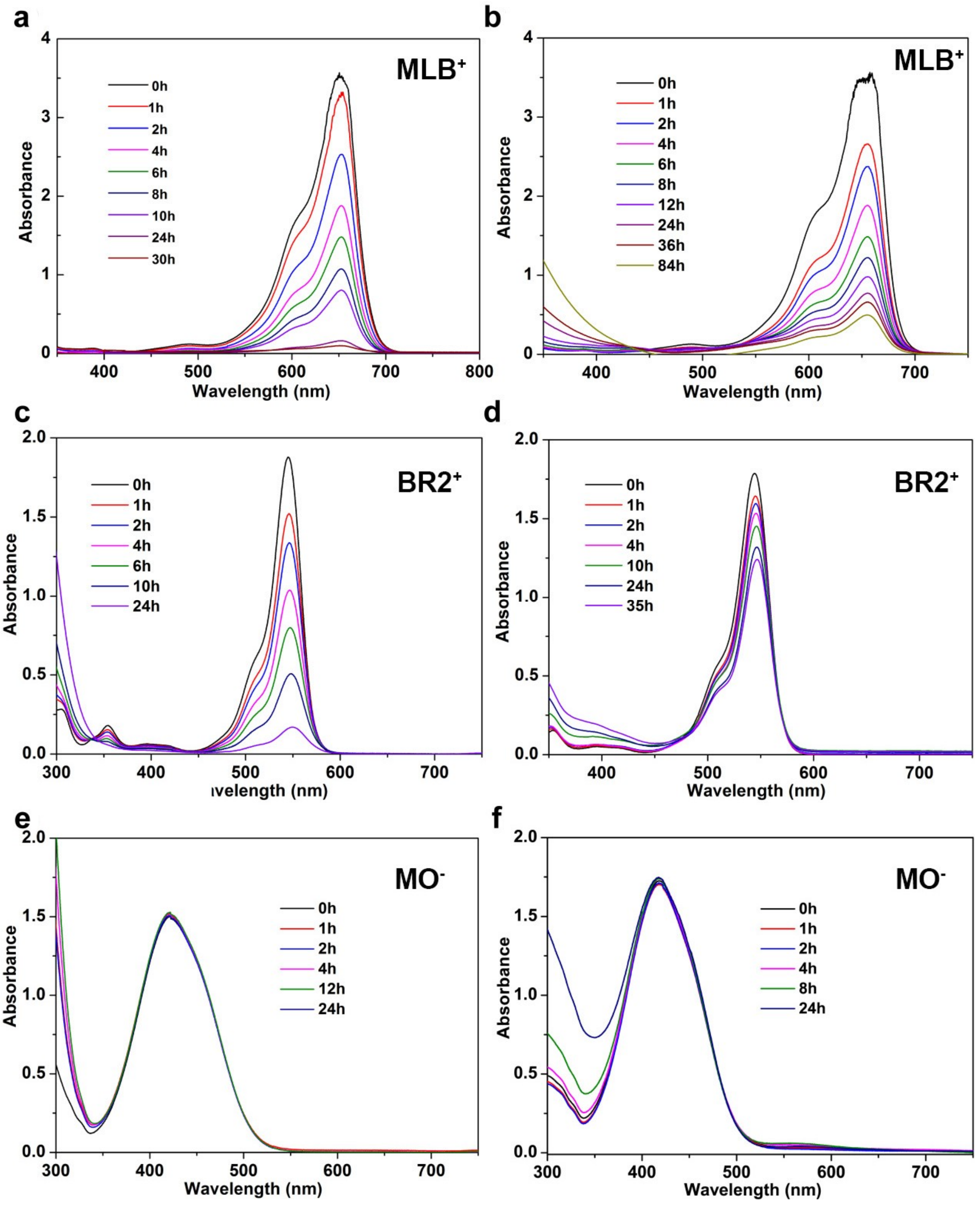

Supplementary Fig. 12. Temporal evolution of UV-Vis absorption spectra of MLB^{+}, $BR2^{+}$ and MO^{-} solution in the presence of **1a** (a,c,e) and **1** (b,d,f).

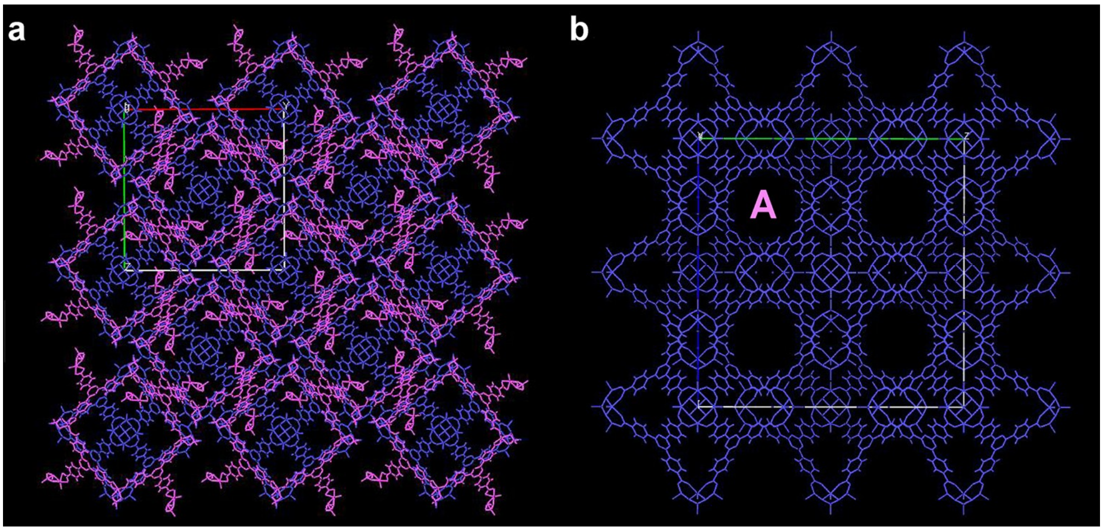

Supplementary Fig. 13. The 3D lattice packing arrangements of **1** (a) and **1a** (b).

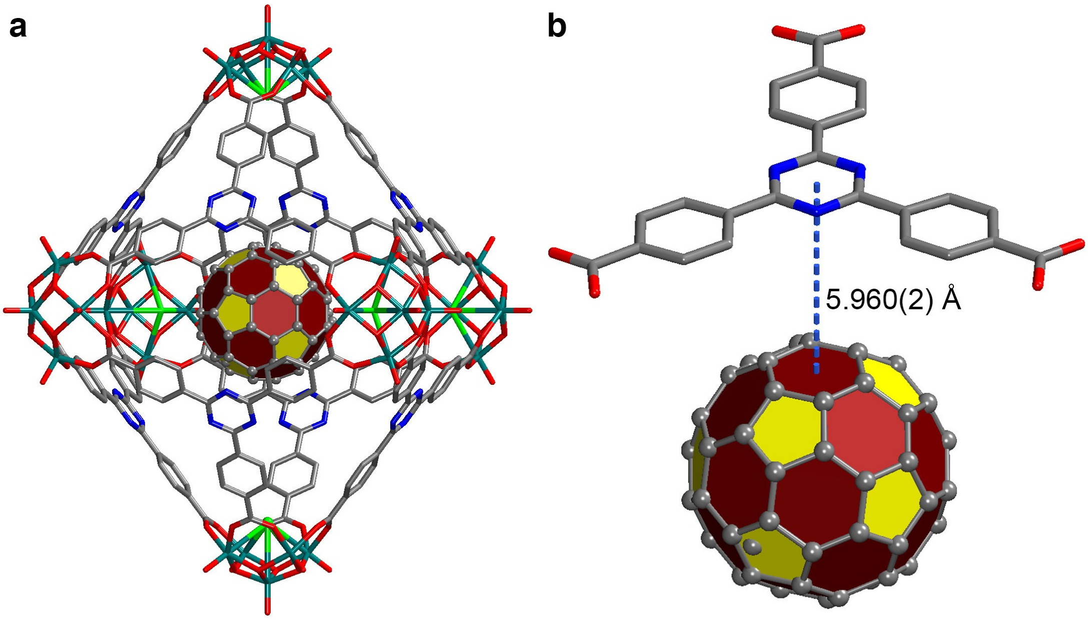

Supplementary Fig. 14. The interaction between C_{60} and **1a** (a), highlighting the completely parallel and no–displaced triazine rings and six-membered rings (b).

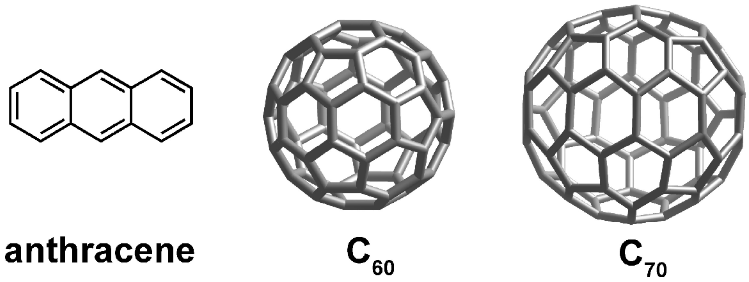

Supplementary Fig. 15. A schematic view of anthracene, C_{60} and C_{70}.

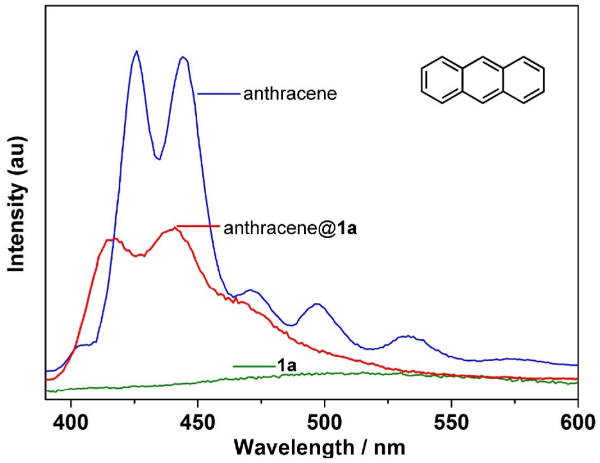

Supplementary Fig. 16. Solid-state emission spectra of anthracene (blue), **1a** (green), and anthracene@**1a** (red).

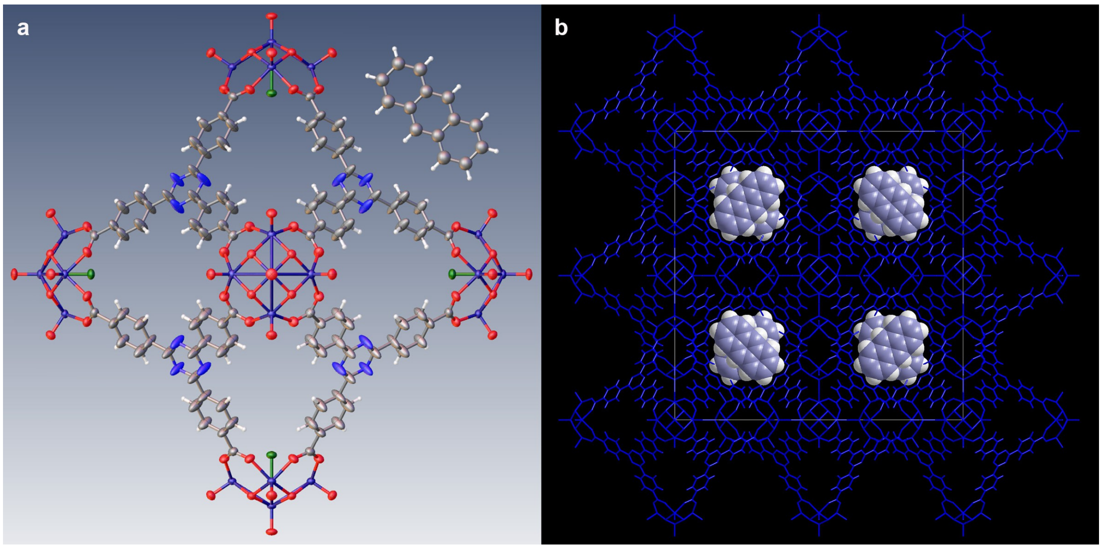

Supplementary Fig. 17. ORTEP diagram (a) and 3D packing arrangement (b) of anthracene@**1a**.

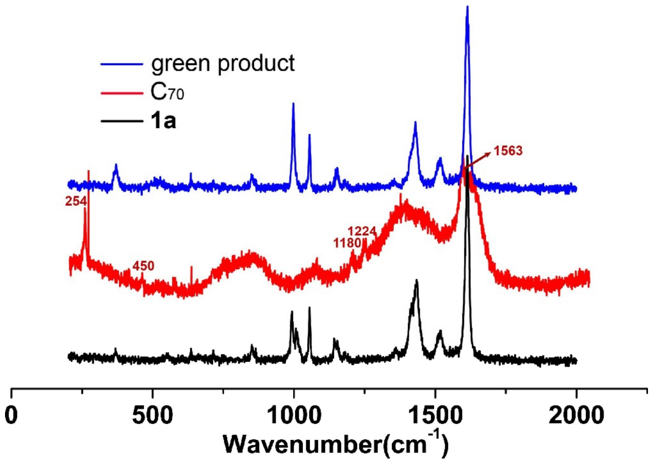

Supplementary Fig. 18. Raman spectra of C_{70}, **1a** and green product after addition of C_{70}.

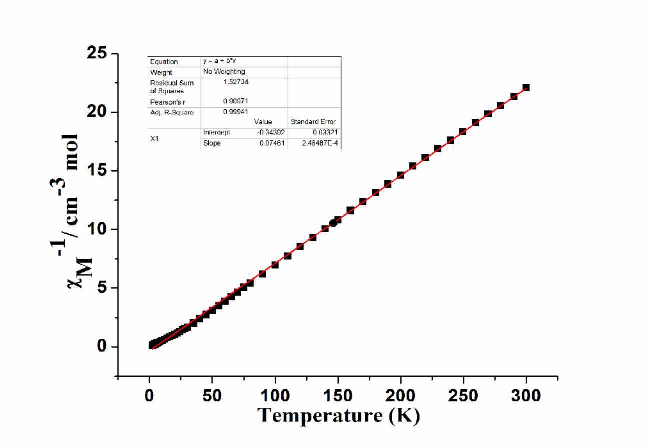

Supplementary Fig. 19. The plot of $1/\chi_M$ versus T for **1**. The red line is the fitting result with $\chi_M = C/(T-\theta)$.

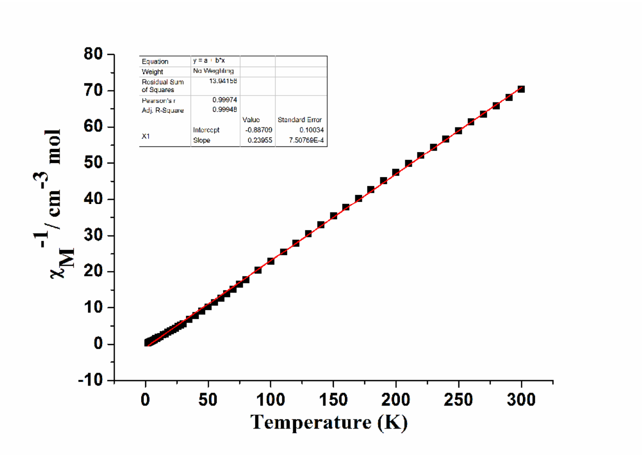

Supplementary Fig. 20. The plot of $1/\chi_M$ versus T for **1a**. The red line is the fitting result with $\chi M = C/(T-\theta)$.

Supplementary References

1. Sudik, A. C., Côté, A. P., Wong-Foy, A. G., O'Keeffe, M., Yaghi, O. M. *Angew. Chem. Int. Ed.* **45**, 2528-2533, (2006)

2. Férey, G., Mellot-Draznieks, C., Serre, C., Millange, F., Dutour, J., Surblé, S., Margiolaki, I. *Science* **309**, 2040-2042, (2005)

3. Zhang, Z., Wojtas, L., Zaworotko, M. J. *Chem. Sci.* **5**, 927–931, (2014)
